# Impact of CLSI and EUCAST breakpoint discrepancies on reporting of antimicrobial susceptibility and AMR surveillance

**DOI:** 10.1016/j.cmi.2019.03.007

**Published:** 2019-07

**Authors:** T.P. Cusack, E.A. Ashley, C.L. Ling, S. Rattanavong, T. Roberts, P. Turner, T. Wangrangsimakul, D.A.B. Dance

**Affiliations:** 1) Lao-Oxford-Mahosot Hospital-Wellcome Trust Research Unit (LOMWRU), Microbiology Laboratory, Mahosot Hospital, Vientiane, Lao People's Democratic Republic; 2) National Infection Service, Public Health England, London, UK; 3) Myanmar Oxford Clinical Research Unit, Yangon, Myanmar; 4) Centre for Tropical Medicine and Global Health, Nuffield Department of Medicine, University of Oxford, Oxford, UK; 5) Shoklo Malaria Research Unit, Mahidol–Oxford Tropical Medicine Research Unit, Faculty of Tropical Medicine, Mahidol University, Mae Sot, Thailand; 6) Cambodia–Oxford Medical Research Unit, Angkor Hospital for Children, Siem Reap, Cambodia; 7) Mahidol–Oxford Tropical Medicine Research Unit, Faculty of Tropical Medicine, Mahidol University, Bangkok, Thailand; 8) Faculty of Infectious and Tropical Diseases, London School of Hygiene and Tropical Medicine, London, UK

To the Editor,

Despite the importance of antimicrobial susceptibility testing (AST) for clinical management of infection and antimicrobial resistance (AMR) surveillance, the methodologies and breakpoints of the two most commonly used systems worldwide, Clinical and Laboratory Standards Institute (CLSI) and European Committee for Antimicrobial Susceptibility Testing (EUCAST), are far from harmonized. Both systems are recommended in the World Health Organization's Global Antimicrobial Resistance Surveillance System (GLASS) [1]), but how discrepancies between the two systems will be addressed is unclear.

Mahidol-Oxford Tropical Health Network (MORU) laboratories in Thailand, Laos, and Cambodia currently use CLSI disc diffusion AST guidelines for routine diagnostic and research purposes, but have recently been considering a switch to EUCAST. As part of a comprehensive review of the practical implications of this, we examined the impact of discrepancies in CLSI and EUCAST zone diameter breakpoints on antimicrobial susceptibility interpretation of frequently isolated Gram-negative organisms at one of our sites, the Microbiology Laboratory, Mahosot Hospital, Vientiane, Laos, in 2017. We also performed a literature search to compare our results to published reports.

The Mahosot Microbiology Laboratory receives clinical samples from Mahosot Hospital and other hospitals within Vientiane and several provincial sites, participates in the United Kingdom National External Quality Assessment (NEQAS) scheme for AST, and is working towards International Organization for Standardization (ISO) 15189 accreditation. Zone diameter data for first-line antimicrobial agents tested according to CLSI standards against all non-duplicate (first isolate per patient) clinical isolates of *Escherichia coli*, *Klebsiella pneumoniae*, and *Pseudomonas aeruginosa* from 1 January 2017 to 31 December 2017 were extracted from the Laboratory Information Management System and interpreted separately using EUCAST 2018 [2] and CLSI 2018 [3] breakpoints as susceptible, intermediate, or resistant and category agreement (percentage of isolates with the same result) determined. These organisms were selected as they were the commonest Gram-negative isolates in 2017 for which both organisations provide clinical breakpoints, and recent studies have reported discrepancies in their susceptibility interpretation [4], [5].

Results are summarized in Table 1. A total of 428 *E. coli*, 208 *K. pneumoniae*, and 78 *P. aeruginosa* isolates were included. Ciprofloxacin resistance rates would have been markedly higher using EUCAST breakpoints (59.1% vs. 46.5% in *E. coli*; 37.5% vs. 13.9% in *K. pneumoniae*; 28.2% vs. 10.3% in *P. aeruginosa*). Resistance to amoxicillin–clavulanic acid among *E. coli* and *K. pneumoniae* would have also increased (52.3% vs. 19.9% and 35.6% vs. 22.1% respectively), reflecting the lower resistance breakpoint and the lack of an intermediate category for this agent in EUCAST Enterobacteriaceae guidelines. Meropenem resistance rates in *P. aeruginosa* would have remained the same, but nine out of 78 (11.5%) isolates would have been reinterpreted from susceptible to intermediate. Category agreement for all other pathogen–antimicrobial combinations would have been >95%.

**Table 1:** Comparison of susceptibilities of *E. coli*, *K. pneumoniae* and *P. aeruginosa* isolated at Mahosot Microbiology Laboratory in 2017 to first-line antibiotics using CLSI and EUCAST criteria

Organism (no. isolates)	Antimicrobial agent	CLSI 2018 (%)			EUCAST 2018 (%)			Category agreement (%)
		S	I	R	S	I	R	
*E. coli* (428)	Amoxicillin-clavulanic acid	55.6	24.5	19.9	47.7	–	52.3	64.7
	Ampicillin	5.8	2.3	91.8	8.2	–	91.8	97.7
	Ciprofloxacin	50.5	3	46.5	31.3	9.6	59.1	77.8
	Gentamicin	58.4	0	41.6	55.1	2.8	42.1	96.7
	Ceftriaxone	41.8	0.2	57.9	40.4	1.4	58.2	98.4
	Trimethoprim-sulphamethoxazole	25.9	0.5	73.6	25.9	0.2	73.8	99.8
*K. pneumoniae* (208)	Amoxicillin-clavulanic acid	67.3	10.6	22.1	64.4	–	35.6	85.6
	Ciprofloxacin	72.6	13.5	13.9	47.6	14.9	37.5	61.5
	Gentamicin	76.4	0	23.6	73.6	2.9	23.6	97.1
	Ceftriaxone	68.8	0	31.3	64.9	3.8	31.3	98.4
	Trimethoprim-sulphamethoxazole	63	2.4	34.6	63.9	0.5	35.6	99.8
*P. aeruginosa*∗ (78)	Ciprofloxacin	85.9	3.8	10.3	71.8	0	28.2	82.1
	Gentamicin	76.9	0	23.1	75.6	0	24.4	98.7
	Meropenem	94.9	0	5.1	83.3	11.5	5.1	88.5

S, susceptible; I, intermediate; R, resistant.

–, no intermediate category.

∗ Ceftazidime not included due to discordant CLSI and EUCAST disc contents.

These results demonstrate that adopting EUCAST breakpoints would have significantly altered susceptibility reporting for two first-line agents tested against each of the most frequently isolated Gram-negative pathogens at Mahosot Microbiology Laboratory in 2017. This artificial change in susceptibility rates, predominantly driven by reclassification of isolates from susceptible to intermediate or resistant, is likely to have influenced antimicrobial selection by clinicians. This would also have distorted AMR surveillance data both locally and nationally given that Mahosot Microbiology Laboratory is one of the few sites in Laos capable of providing AMR data. *Escherichia coli*-ciprofloxacin and *K. pneumoniae*-ciprofloxacin are GLASS priority pathogen–antimicrobial combinations, but category agreement was only 77.8% and 61.5% respectively between EUCAST and CLSI. It must be noted that updated CLSI ciprofloxacin breakpoints for Enterobacteriaceae and *P. aeruginosa* are more closely aligned with EUCAST, meaning some but not all discrepancies in ciprofloxacin susceptibility would have been eliminated had 2019 breakpoints [6], [7] been used for the analysis.

Our results are supported by a literature search (see Fig. S1, Table S1) which identified 20 articles whose main objective was comparing susceptibility interpretation between CLSI and EUCAST. Nineteen out of 20 articles reported significant discrepancies in one or more pathogen–antimicrobial combination, nearly always due to a reduction in susceptibility rates and/or increase in resistance rates when applying more restrictive EUCAST breakpoints. A notable exception was a study from India [8] that reported higher meropenem and imipenem susceptibility rates in urinary *E. coli* isolates using EUCAST breakpoints, underlining the lack of alignment between the two systems even for such important combinations. A trend of reduced susceptibility and/or higher resistance rates with EUCAST breakpoints was evident in the additional 53 articles where both EUCAST and CLSI breakpoints were used for susceptibility interpretation, many of which were reports of AMR surveillance data.

In conclusion, discrepancies in clinical breakpoints between CLSI and EUCAST significantly impact susceptibility interpretation of clinical isolates, with generally lower susceptibility rates when EUCAST guidelines are used. This has implications not only for antibiograms at institutions switching between the two AST systems, but for broader AMR surveillance initiatives comparing data within and between countries using different systems or over the time period during which a change in methodology is implemented. Globally harmonized clinical breakpoints are urgently needed.

## Transparency declaration

No payment or services from a third party for any aspect of the submitted work were received by any of the authors or their institutions. None of the authors have financial relationships with entities in the bio-medical arena that could be perceived to influence what is written in this manuscript. None of the authors have planned, pending or issued patents broadly relevant to the work, and no authors have other relationships/conditions/circumstances that present a potential conflict of interest. The MORU Tropical Health Network is core funded by Wellcome (grant number 106698/Z/14/Z). The funding body had no role in the design of the study and collection, analysis, and interpretation of data and writing in the manuscript.
