# Supplementary material for: Impact of CLSI and EUCAST breakpoint discrepancies on reporting of antimicrobial susceptibility and AMR surveillance
Source: Clin Microbiol Infect. 2019 Jul;25(7):910–1. doi: 10.1016/j.cmi.2019.03.007 (PMC6587648; doi:10.1016/j.cmi.2019.03.007)
Supplement: Multimedia component 1 [file mmc1.pdf]

Supplementary Material

Supplementary Figure S1. Literature search flow diagram

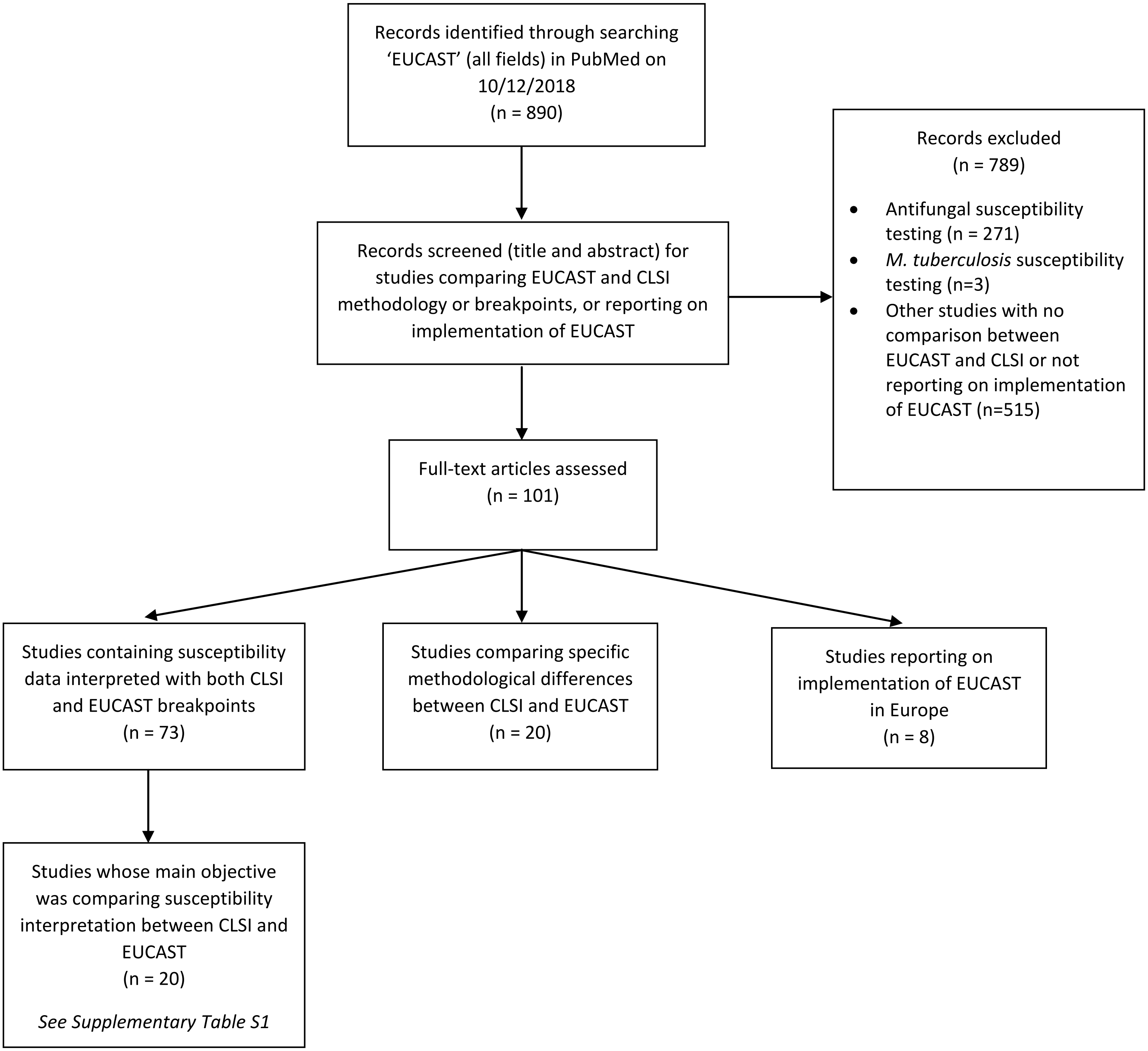

Supplementary Table S1. Summary of articles whose main objective was comparing susceptibility interpretation between CLSI and EUCAST

Author and Year (Pubmed ID)	Origin of isolates	CLSI breakpoints used	EUCAST breakpoints used	AST method	Main discrepancies when EUCAST breakpoints applied
Bork 2017 (29031525)	USA	2014	2011	MIC	Reduced cefepime susceptibility in Enterobacteriaceae
Cagan Aktas 2014 (25492650)	Turkey	2011	2011	MIC	Similar fosfomycin susceptibility in urinary *E. coli*
Hawser 2010 (20598511)	Global	2010	2010	MIC	Reduced susceptibility to ceftazidime in ESBL-producing *E. coli*
Hombach 2012 (22167240)	Switzerland	2011	2011	DD	Increased cefepime resistance in Enterobacteriaceae, increased cefepime and meropenem resistance in *P. aeruginosa*, increased tobramycin and gentamicin resistance in *A. baumannii*
Hombach 2013 (23633681)	Switzerland	2013	2013	DD	Reduced susceptibility to ceftazidime and cefepime in ESBL and AmpC-producing Enterobacteriaceae
Hombach 2013 (23596246)	Switzerland	2009	2011	DD	Increased multi-drug resistance rates in Enterobacteriaceae and *P. aeruginosa*
Jones 2013 (23514756)	USA, Europe, S. America, Asia-Pacific	2013	2013	MIC	Reduced minocycline susceptibility in methicillin-resistant *S. aureus*
Jones 2013 (23490012)	USA, Europe, S. America, Asia-Pacific	2013	2013	MIC	Slight increase in doxycycline susceptibility in *S. pneumoniae*; slight decrease in tetracycline and doxycycline susceptibility in *S. pyogenes* and *S. aureus*
Kassim 2016 (27068515)	Kenya	2015	2015	MIC	Reduced co-amoxiclav, nitrofurantoin, amikacin, ceftazidime, cefepime susceptibility in *E. coli*; reduced gentamicin susceptibility in *S. aureus*; reduced amikacin and ciprofloxacin susceptibility in *P. aeruginosa*
Machuca 2016 (25772329)	Spain	2014	2014	MIC	Reduced fluoroquinolone susceptibility in *E. coli* harbouring fluoroquinolone resistance genes

Marchese 2012 (22866984)	Europe and USA	2012	2012	MIC	Reduced levofloxacin susceptibility in *S. pyogenes*; reduced benzylpenicillin, co-amoxiclav and cefaclor susceptibility in *S. pneumoniae*; reduced cefaclor and cefuroxime susceptibility in *H. influenzae* and *M. catarrhalis*
O'Halloran 2018 (29210602)	Ireland	2017	2017	DD	Reduced susceptibility to co-amoxiclav and ciprofloxacin in urinary *E. coli*
Rodriguez-Bano 2012 (21985560)	Spain	2010	2011	MIC	Reduced susceptibility to ceftazidime, cefepime, amikacin in ESBL-producing *E. coli*
Rodríguez-Martínez 2011 (21427105)	Spain	2010	2010	MIC	Reduced fluoroquinolone susceptibility in *E. coli* harbouring fluoroquinolone resistance genes
Sahu 2018 (30078964)	India	2016	2016	MIC	Higher meropenem and imipenem susceptibility in urinary *E. coli*
Sanchez-Bautista 2018 (28479139)	Spain	2017	2017	MIC	Reduced susceptibility to clindamycin, tetracycline, gentamicin and tobramycin in *S. aureus*; reduced susceptibility to tobramycin and norfloxacin in *E. coli*; reduced susceptibility to amikacin, fosfomycin and norfloxacin in *K. pneumoniae*; reduced susceptibility to amikacin, aztreonam, ciprofloxacin and levofloxacin in *P. aeruginosa*
Schito 2010 (21123159)	Europe and Brazil	2009	2010	MIC	Higher ampicillin and co-amoxiclav resistance and higher cefuroxime susceptibility in *E. coli*
Suzuk 2015 (26649407)	Turkey	2014	2014	DD	Reduced susceptibility to gentamicin and levofloxacin, increased susceptibility to cefuroxime axetil in urinary *E. coli* isolates
van der Bij 2012 (22925456)	Holland	2009	2010	MIC	Reduced susceptibility to ciprofloxacin, ceftazidime, cefotaxime/ceftriaxone in *E. coli*
Wolfensberger 2013 (24223893)	Switzerland	2013	2013	DD	Reduced cefepime susceptibility in Enterobacteriaceae and reduced meropenem susceptibility in *P. aeruginosa*

***DD** = disk diffusion method, **MIC** = Minimum Inhibitory Concentration method*

Supplementary Table S1 References

1. Bork JT, Heil EL, Leekha S, Fowler RC, Hanson ND, Majumdar A et al. Impact of CLSI and EUCAST Cefepime breakpoint changes on the susceptibility reporting for Enterobacteriaceae. Diagn Microbiol Infect Dis. 2017 Dec;89(4):328-333. PMID: 29031525
2. Cagan Aktas S, Gencer S, Batirel A, Haciseyitoglu D, Ozer S. Fosfomycin susceptibility of urinary Escherichia coli isolates producing extended-spectrum beta-lactamase according to CLSI and EUCAST recommendations. Mikrobiyol Bul. 2014;48:545–555. PMID: 25492650
3. Hawser SP, Badal RE, Bouchillon SK, Hoban DJ, Hsueh PR. Comparison of CLSI 2009, CLSI 2010 and EUCAST cephalosporin clinical breakpoints in recent clinical isolates of *Escherichia coli*, *Klebsiella pneumoniae* and *Klebsiella oxytoca* from the SMART Global Surveillance Study. Int J Antimicrob Agents. 2010;36(3):293–4. PMID: 20598511
4. Hombach M, Bloemberg GV, Bottger EC. Effects of clinical breakpoint changes in CLSI guidelines 2010/2011 and EUCAST guidelines 2011 on antibiotic susceptibility test reporting of Gram-negative bacilli. The Journal of antimicrobial chemotherapy. 2012;67(3):622-32. PMID: 22167240
5. Hombach M, Mouttet B, Bloemberg GV. Consequences of revised CLSI and EUCAST guidelines for antibiotic susceptibility patterns of ESBL- and AmpC β-lactamase-producing clinical *Enterobacteriaceae* isolates. J. Antimicrob. Chemother. 2013;68 2092–2098. PMID: 23633681
6. Hombach M, Wolfensberger A, Kuster SP, Bottger EC. Influence of clinical breakpoint changes from CLSI 2009 to EUCAST 2011 AST guidelines on multidrug resistance rates of Gram-negative rods. J. Clin. Microbiol. 2013;51:2385–2387. PMID: 23596246
7. Jones RN, Wilson ML, Weinstein MP, Stilwell MG, Mendes RE. Contemporary potencies of minocycline and tetracycline HCL tested against Gram-positive pathogens: SENTRY Program results using CLSI and EUCAST breakpoint criteria. Diagn Microbiol Infect Dis. 2013;75:402–5. PMID:
8. Jones RN, Stilwell MG, Wilson ML, Mendes RE. Contemporary tetracycline susceptibility testing: doxycycline MIC methods and interpretive criteria (CLSI and EUCAST) performance when testing Gram-positive pathogens. Diagn Microbiol Infect Dis. 2013;76:69–72. PMID: 23490012
9. Kassim A, Omuse G, Premji Z, Revathi G. Comparison of clinical laboratory standards institute and European committee on antimicrobial susceptibility testing guidelines for the interpretation of antibiotic susceptibility at a university teaching hospital in Nairobi, Kenya: A cross-sectional study. Ann Clin Microbiol Antimicrob. 2016;15:21. PMID: 27068515
10. Machuca J, Briales A, Díaz-de-Alba P, Martínez-Martínez L, Rodríguez-Martínez JM, Pascual Á. Comparison of clinical categories for Escherichia coli harboring specific qnr and chromosomal-mediated fluoroquinolone resistance determinants according to CLSI and EUCAST. Enferm Infecc Microbiol Clin. 2016 Mar;34(3):188-90. PMID: 25772329
11. Marchese A, Esposito S, Barbieri R, Bassetti M, Debbia E. Does the adoption of EUCAST susceptibility breakpoints affect the selection of antimicrobials to treat acute community-acquired respiratory tract infections? BMC Infect Dis. 2012;12:181. PMID: 22866984

12. O'Halloran C, Walsh N, O'Grady MC, Barry L, Hooton C, Corcoran GD et al. Assessment of the comparability of CLSI, EUCAST and Stokes antimicrobial susceptibility profiles for Escherichia coli uropathogenic isolates. Br J Biomed Sci. 2018;75(1):24-29. PMID: 29210602
13. Rodriguez-Bano J, Picon E, Navarro MD, Lopez-Cerero L, Pascual A. Impact of changes in CLSI and EUCAST breakpoints for susceptibility in bloodstream infections due to extended-spectrum beta-lactamase-producing Escherichia coli. Clin Microbiol Infect. 2012;18: 894–900. PMID: 21985560
14. Rodriguez-Martínez J. M., Briales A., Velasco C., Diaz de Alba P., Martínez-Martínez L., Pascual A. Discrepancies in fluoroquinolone clinical categories between the European Committee on Antimicrobial Susceptibility Testing (EUCAST) and CLSI for *Escherichia coli* harbouring qnr genes and mutations in gyrA and parC. J. Antimicrob. Chemother. 2011;66, 1405–1407. PMID: 21427105
15. Sahu C, Jain V, Mishra P, Prasad KN. Clinical and laboratory standards institute versus European committee for antimicrobial susceptibility testing guidelines for interpretation of carbapenem antimicrobial susceptibility results for Escherichia coli in urinary tract infection (UTI). J Lab Physicians. 2018 Jul-Sep;10(3):289-293. PMID: 30078964
16. Sanchez-Bautista A, Coy J, Garcia-Shimizu P, Rodriguez JC. From CLSI to EUCAST guidelines in the interpretation of antimicrobial susceptibility: What is the effect in our setting? Enferm Infecc Microbiol Clin. 2018;36(4):229-232. PMID: 28479139
17. Schito GC, Gualco L, Naber KG, Botto H, Palou J, Mazzei T et al. Do different susceptibility breakpoints affect the selection of antimicrobials for treatment of uncomplicated cystitis? J Chemother. 2010;22:345–354. PMID: 21123159
18. Süzük S, Kaşkatepe B, Avcıküçük H, Aksaray S, Başustaoğlu A. [The comparison of antibiotic susceptibilities of uropathogenic *Escherichia coli* isolates in transition from CLSI to EUCAST] Mikrobiyol Bul. 2015;49:494–501. PMID: 26649407
19. van der Bij AK, van Dijk K, Muilwijk J, Thijsen SF, Notermans DW, de Greeff S, et al. Clinical breakpoint changes and their impact on surveillance of antimicrobial resistance in Escherichia coli causing bacteraemia. Clin Microbiol Infect. 2012;18(11):E466-72. PMID: 22925456
20. Wolfensberger A, Sax H, Weber R, Zbinden R, Kuster SP, Hombach M. Change of antibiotic susceptibility testing guidelines from CLSI to EUCAST: influence on cumulative hospital antibiograms. PloS one. 2013;8(11):e79130. PMID: 24223893
